# Evaluation of oxidative stress in seminal plasma of adolescents with varicocele

**DOI:** 10.1530/RAF-20-0048

**Published:** 2021-05-28

**Authors:** Valéria Barradas, Mariana Pereira Antoniassi, Paula Intasqui, Marcilio Nichi, Ricardo Pimenta Bertolla, Deborah Montagnini Spaine

**Affiliations:** 1 Department of Surgery, Division of Urology, Human Reproduction Section, Universidade Federal de São Paulo, São Paulo, Brazil; 2 Hospital São Paulo, São Paulo, Brazil; 3 Department of Animal Reproduction, School of Veterinary Medicine and Animal Sciences, University of Sao Paulo, São Paulo, Brazil

**Keywords:** varicocele, oxidative stress, antioxidants, adolescent, seminal analysis

## Abstract

Varicocele, defined by a dilation of efferent testicular veins, is the most commonly identifiable, surgically correctable lesion associated with male-factor infertility, starts at puberty and causes a progressive decline in fertility potential. The pathophysiology of infertility caused by this disease is still poorly understood, but it is suggested that the main mechanism is oxidative stress. Therefore, the aim of this study was to verify if the varicocele is associated with changes in enzymatic antioxidant mechanisms and seminal plasma lipid peroxidation levels in adolescents. We recruited 90 adolescents that were divided into control (C; *n*  = 27); varicocele and normal semen (VNS; *n*  =46); varicocele and altered semen (VAS; *n*  =17). Seminal and serum levels of lipid peroxidation were quantified by thiobarbituric acid reactive substances (TBARS). Seminal plasma antioxidant profile was evaluated by the activities of catalase (CAT), glutathione peroxidase (GPx) and superoxide dismutase (SOD). The VAS group had increased lipid peroxidation levels when compared to the other groups. The levels of serum lipid peroxidation and activities of the enzymes SOD and GPx did not differ between groups. CAT was undetectable by the method used. In conclusion, in adolescents with varicocele and altered semen analysis, there is an increase in seminal lipid peroxidation levels compared to adolescents with varicocele and without seminal change and adolescents without evident varicocele. However, the observed oxidative stress is not caused by a decrease in superoxide dismutase and glutathione peroxidase activities, which did not differ between adolescents with and without evident varicocele.

**Lay summary:**

Varicocele, defined by a dilation of efferent testicular veins, is the most commonly identifiable, surgically correctable lesion associated with male-factor infertility, starts at puberty and causes a progressive decline in fertile potential. There is still much that is not understood regarding how exactly it affects semen quality, but most studies agree that oxidative stress, which is defined as excessive amounts of free radicals in relation to antioxidant defense, is an important mechanism. In this study, we aimed to verify if the varicocele is associated with changes in antioxidant defense and semen oxidation in 90 adolescents with and without varicocele. In adolescents with varicocele and abnormal semen, there is an increase in semen oxidation compared to controls or to the group with varicocele and normal semen quality. Our results can help to understand how varicocele leads to infertility in adolescents, identifying changes in oxidative activity in semen, since the onset of varicocele and before damage to sperm production can be detected.

## Introduction

Infertility, defined as the couple's failure to conceive after 12 months of regular and unprotected sexual intercourse, affects about 15% of couples in reproductive age and the male factor is associated in up to 50% of cases (Brugh & Lipshultz 2004). Varicocele is the main treatable cause of male infertility and is characterized by an abnormal dilation of the pampiniform plexus veins, accompanied by a reversal of blood flow and by venous stasis (Brugh *et al.* 2003). Its prevalence increases gradually until the age of 15, when it reaches the same prevalence observed in adults: approximately 15% of the male population (Gorelick & Goldstein 1993, Witt & Lipshultz 1993). Moreover, its prevalence can reach about 35–40% in primary infertility and 80% in secondary infertility, suggesting a progressive impairment in the male fertile potential caused by varicocele (Clavijo *et al.* 2017).

The mechanism of varicocele associated with infertility has not yet been fully understood. However, in order to explain the impaired spermatogenesis commonly observed in these patients, some complementary and interconnected hypotheses have been postulated: (i) an increase in scrotal and testicular temperature; (ii) presence of testicular hypoxia; (iii) presence of reflux in kidney and adrenal metabolites; (iv) decreased perfusion of the affected testicle caused by increased venous pressure; and (v) hormonal dysfunction (Jensen *et al.* 2017).

The venous stasis caused by varicocele decreases blood flow in the testis, impairing the arterial blood cooling system, thus leading to testicular hyperthermia. Therefore, the metabolism of germ cells is accelerated during spermatogenesis, which, in turn, is not accompanied by an increase in testicular blood supply. Thus, the absence of adequate oxygenation induces a chronic hypoxia state, which causes a direct damage to germ cells as well as activates reactive oxygen species (ROS)-generating metabolic pathways (Hassanin *et al.* 2018).

An excess in ROS levels is harmful to Leydig, Sertoli and spermatogenic cells, and has a negative impact on several functions of mature sperm (Cho *et al.* 2016). Furthermore, ROS are able to destabilize the double bonds of polyunsaturated fatty acids (PUFAs), the main component of sperm and mitochondrial membranes, in a chain reaction known as lipid peroxidation. Once established, this process becomes autocatalytic, leading to the formation of several highly toxic metabolites, mainly malondialdehyde (MDA), which has been widely used as a marker of lipid peroxidation (Benedetti *et al.* 2012, Tsikas 2017). Lipid peroxidation is responsible for serious alterations in sperm function, such as mitochondrial dysfunction and reduced sperm motility and ability to undergo acrosome reaction, in addition to alterations to the sperm morphology (Aitken 2020).

Semen antioxidant defense systems, present in both sperm and seminal plasma, can counteract ROS excessive levels. Under physiological conditions, there is a balance between ROS production and the antioxidant protection. However, when an imbalance occurs in favor of the oxidant, due to a reduction in the antioxidant capacity and/or to increased ROS production, oxidative stress occurs (Alahmar 2019). Sperm are especially susceptible to oxidative stress due to their low cytoplasmic content, insufficient for antioxidant protection, and the high concentration of PUFAs in their plasma membrane, which makes them highly dependent on the antioxidant machinery in the seminal plasma (Aitken *et al.* 2012). Therefore, the seminal plasma has an important role in supporting sperm function, protecting the male gametes from ROS damaging effects (Szczykutowicz *et al.* 2019). The antioxidant activity of superoxide dismutase (SOD), catalase (CAT) and glutathione reductase (GPx) protects the sperm membrane against lipid peroxidation in an attempt to preserve its integrity. Thus, the seminal activity of these enzymes associated with MDA levels has been described as effective indicators of oxidative stress (Agarwal & Majzoub 2017).

Previous studies have shown that young men with varicocele have high ROS levels and sperm lipid peroxidation levels (Romeo *et al.* 2003; Türkyilmaz *et al.* 2004). However, it remains to be elucidated how oxidative stress occurs in the seminal plasma of adolescents with varicocele and affects sperm function. Thus, the objective of our study was to determine whether, in adolescents, varicocele is associated with alterations in the enzymatic antioxidant mechanisms and lipid peroxidation levels in the seminal plasma.

## Methods

### Study design

A cross-sectional study was conducted with 128 adolescents, recruited from the National Service of Industrial Learning/SENAI 'Conde José Vicente de Azevedo' in Sao Paulo, Brazil. The adolescents and their legal guardians were invited to a lecture, and those who agreed to proceed in the study signed informed consent (their own and their legal guardian’s). Institutional Review Board approval was obtained from the Sao Paulo Federal University Ethics Committee.

Medical interview and genital physical examination were performed in all adolescents by the same urologist. Varicocele was diagnosed by scrotal palpation, with the adolescent in the standing position and in a controlled temperature environment (23–25°C), according to the American Society for Reproductive Medicine (ASRM) guidelines (Practice Committee of the American Society for Reproductive Medicine and Society for Male Reproduction and Urology 2014).

Semen samples of all adolescents were collected by masturbation after ejaculatory abstinence of 2–7 days. After semen liquefaction, the sample was divided into two aliquots. The first was used for semen analysis, according to the World Health Organization (WHO) criteria (2010) (World Health Organization, Department of Reproductive Health and Research 2010). Semen volume was assessed with the aid of a serological pipette and after pH testing strips were used. Motility was assessed manually by Horwell (Arnold R. Horwell Limited®, London) chamber, and the sperm were classified as progressive, non-progressive and immotile. Vitality was performed in samples which the progressive motility and non-progressive were less than 40%; semen aliquots were stained with eosin-nigrosin (Sigma-Aldrich) and classified as alive (white spermatozoa) and dead (pink spermatozoa). Sperm concentration was assessed by a modified Neubauer chamber (Herka®, Germany), and the result was expressed in millions per milliliter. For morphological evaluations, the kit Panotico (Laborclin®, Brazil) was used to stained sperm smears, and the Kruger’s criteria was used. Round cells were determined using a modified Neubauer chamber (Herka®, Germany) in semen samples diluted in 0.9% saline, and leukocytes were detected by peroxidase test. All analyses were performed by one experienced technician. The second aliquot was centrifuged at 16,000 ***g*** for 15 min to separate the seminal plasma from the cellular debris. The seminal plasma was then frozen and stored at −20°C without cryoprotectants until lipid peroxidation and antioxidants activity analyses. All adolescents collected two samples, with a minimum interval of 7 days and maximum of 15 days between collections. Venous blood samples were also collected from all adolescents after the first semen collection and were centrifuged at 300 ***g*** for 10 min. The serum was then frozen at −20°C until lipid peroxidation analysis.

Inclusion criteria were male adolescents aged between 15 and 17 years, with full sexual maturity (Tanner stage V) who have already initiated the practice of masturbation. Exclusion criteria were: fever reported in the 90 days prior to semen collection; obesity or overweight; smoking, drinking or drug habits; presence or history of systemic diseases (and their treatments); genital malformation; genetic syndrome; testicular dystopia; and history of inguinal-scrotal surgery, orchitis, epididymitis or torsion of the spermatic cord. Adolescents presenting semen volume below 0.5 mL, leukocitospermia and/or azoospermia, or those without varicocele, but with altered semen analysis were also excluded. Due to the applied exclusion criteria, 38 adolescents were excluded from the study.

Based on the presence of varicocele and on semen analysis, the 90 adolescents were further divided into 3 groups: (i) control group, composed by 27 adolescents without varicocele or with varicocele grade I, without any alteration in semen, (ii) varicocele and normal semen (VNS), with 46 adolescents presenting with varicocele grades II and/or III and without alterations in semen, and (iii) varicocele and altered semen (VAS), consisting of 17 adolescents with varicocele grades II and/or III and at least one alteration in semen. Control group included men with varicocele grade I, because our group has previously demonstrated that semen variables and sperm function of adolescents with varicocele grade I do not differ from those of adolescents without varicocele (Mori *et al.* 2008).

### Lipid peroxidation evaluation in seminal plasma

Semen lipid peroxidation was determined using the method previously described by Ohkawa *et al.* for the determination of lipid peroxidation products, mainly MDA (Ohkawa *et al.* 1979). The method is based on the reaction of two molecules of thiobarbituric acid with one molecule of malondialdehyde, at high temperatures and low pH, resulting in a pink chromogen that can be quantified with a spectrophotometer. Briefly, 500 µL of seminal plasma were added to 1000 µL of 10% (w:v in water) trichloroacetic acid (TCA) and centrifuged at 16,000 ***g*** for 15 min at 15°C for protein removal. After, 500 µL of the supernatant was added to 500 µL of 1% (v:v in 0.05 M sodium hydroxide) TBA in another tube, which was maintained for 10 min in boiling water (90−100°C). Thereafter, the samples were cooled on ice to stop the chemical reaction. The TBARS molecules were quantified by a spectrophotometer (Ultrospec 3300pro, Healthcare® GE) at a wavelength of 532 nm. Results were compared to a standard curve previously prepared with a standard solution of malondiadehyde. The TBARS concentration was determined using the value of 1.56 × 10^5^/M cm as the MDA extinction coefficient. Lipid peroxidation in the seminal plasma was described as TBARS in nanograms/milliliter of seminal plasma. The TBARS values were also normalized by the sperm concentration, because the sperm is the major source of MDA production in semen. Therefore, the TBARS (ng/mL) was divided by the concentration of sperm (10^6^/mL) to obtain the TBARS/10^6^ sperm values (Lacerda *et al.* 2011, Sposito *et al.* 2017).

### Evaluation of lipid peroxidation in serum

Lipid peroxidation levels in serum were determined using a modified protocol from Ohkawa et al (Ohkawa *et al.* 1979). Thus, 200 µL of serum were added to 400 µL of a solution containing: 10% (v:v) TFA , 0.375% (v:v) TBA , and 0.25N hydrochloric acid (HCl).

After homogenization, the samples were placed in a water bath (Scientific® Fisher, Isotemp 205) at 100°C for 15 min. Then, the samples were cooled in an ice bath at 0°C for 5 min, and 500 µL of *n* -butanol (Synth®, A1077.01) were added. After homogenization in vortex, the samples were centrifuged (Eppendorf 5804R) for 15 min at 2500 ***g***. The supernatant was collected and transferred in duplicate to a 96-well microplate. TBARS molecules were then quantified by spectrophotometry (ELx800 Absorbance Microplate Reader, Biotek, Vermont, USA) at a wavelength of 532 nm. Results were compared to a standard curve previously prepared with a standard solution of malondiadehyde. Lipid peroxidation levels were described in TBARS nanogram per milliliter of serum.

## Antioxidant enzymes activity in seminal plasma

### Catalase activity

Catalase activity was assessed indirectly by hydrogen peroxide (H_2_O_2_) consumption (Nichi *et al.* 2006). In a quartz cuvette (Biocell, California, USA), 5 µL of seminal plasma were mixed to 20 µL of milli-Q water (Millipore Corporation), 25 µL of TE buffer (50 mM trisaminomethane and 25 mM EDTA, pH 8.0) and 450 µL of 900 µM hydrogen peroxide. Samples were then maintained at 30°C for 5 min, and the absorbance was measured every 5 s, and the curve of H_2_O_2_ consumption was compared to a blank. Samples were read in a spectrophotometer (Ultrospec 3300pro, Healthcare® GE) and calculations considered 0.071/M cm as the molar extinction coefficient of hydrogen peroxide. Catalase activity was calculated based on the formula CAT = (initial absorbance − final)/0.071 × dilution. Results were expressed as UI/mL.

### Glutathione peroxidase (GPx) activity

GPx enzyme activity was assessed indirectly by measuring the NAD phosphate (NADPH) consumption, using the Beutler protocol modified by Bittencourt (de Bittencourt *et al.* 1998). This method is based on measuring the consumption of NADPH; the reaction between a hydroperoxide and reduced glutathione (GSH) that is catalyzed by the GSPH-Px together with the enzyme glutathione reductase (GSSGr) is induced. This reaction causes the conversion of glutathione disulfide (GSSH – glutathione oxidized) to GSH, which in turn consumes NADPH (measured with a spectrophotometer). Briefly, 100 µL of seminal plasma were mixed to 20 µL of 0.25 mM sodium azide, 20 mL of 0.25 UI GSSGr, 100 mL of 0.12 mM GSH, 660 µL of a solution containing 143 mM sodium phosphate and 6.3 mM EDTA, and 1000 µL of 0.12 mM NADPH. Then, the cuvette was stirred in vortex and maintained in the spectrophotometer (Ultrospec 3300pro, Healthcare® GE) for 300 s to stabilize the reaction. After, 100 mL of tert-butyl hydroperoxide in 1.2 mM hydroperoxide (TBHP) were added. The consumption curve of NADPH was measured in a wavelength of 340 nm for 10 min at 37°C (measured at every 5 s) and compared to a blank. Calculations considered the value of 0.0062/M cm as the molar extinction coefficient of NADPH. Glutathione peroxidase activity was calculated based on the formula: GPx activity = (final absorbance − initial)/0.0062 × dilution. Results were expressed as UI/mL.

### Superoxide dismutase (SOD) activity

Superoxide dismutase was indirectly assessed by the rate of cytochrome c reduction by superoxide anion (O_2_−) (Flohé & Otting, 1984). The xanthine–xanthine oxidase system continuously generated O^2−^ (that reduced cytochrome C). The SOD present in the sample competed with the cytochrome C by converting the superoxide free radical to H_2_O_2_ and O^2−^, thereby slowing the rate of cytochrome C reduction. The reaction medium was composed by 1 mM cytochrome c, 50 mM xanthine, 100 mM EDTA and 50 µM sodium phosphate buffer (pH 7.8). The adjustment by the required activity of xanthine oxidase (Sigma-Aldrich®) for the generation of O_2_^−^ and for the reduction of cytochrome c was performed as 0.025 absorbance units per minute, because 1 unit of total SOD activity corresponds to 50% of this value. The assay was performed at 550 nm and 25°C for 5 min. Therefore, SOD activity in the sample decreased the rate of cytochrome reduction when compared to the blank. To calculate SOD activity, we used [(Δ absorbance of sample − 0.025)/0.0125) × dilution]. The results were expressed as U/mL.

### Statistical analysis

Data analysis was performed using the SPSS 18.0 for Windows ® (SPSS). Initially, normality of data distribution was evaluated by Kolmogorov–Smirnov test. Non-normally distributed data were transformed to their logarithmic values.

Groups were compared by ANOVA followed by* post hoc* Tukey HSD. Transformed data that still did not obey the normality assumption were compared using the Kruskal–Wallis test, followed by* post hoc* Tamhane. For these variables, data were presented as median and interquartile range. For correlation analysis, we used the Pearson test (variables that did not show normality were previously transformed). For the entire study, an α of 5% was adopted. Effect size and power of the study were calculated using G*Power 3.1.9.4.

## Results

### Study groups and excluded samples

We recruited 128 adolescents, of which 38 were excluded due to the exclusion criteria (samples that would add bias to our study): 8 with inguinal herniorrhaphy, 8 with semen alteration without varicocele, 7 with leukocytospermia, 3 with semen volume less than 0.5 mL, 2 with obesity, 2 with testicular asymmetry, 1 with overweight, 1 with low testicular volume, 1 with cryptorchidism, 1 with hydrocele, 2 with azoospermia, 1 with orchiepididymitis and 1 with testicular asymmetry+ inguinal herniorrhaphy+ leukocytospermia. Therefore, groups were formed as 27 adolescents in control, 46 in varicocele normal semen and 17 varicocele altered semen. Despite the number of patients be 27, 46 and 17, respectively, when we calculate the effect size and the power of the study, we observe that the effect size is intermediate (Cohen's d = 0.66). Therefore, there is a high magnitude of the real difference between groups. Furthermore, the power observed is 80%. Thus, there is a high chance of the sample to detect a difference that we hope to observe in the population.

### Seminal quality and clinical characteristics

No differences between the groups were observed regarding age (*P* = 0.984), right and left testicular volumes (*P* = 0.363 and *P* = 0.168), ejaculatory abstinence length (*P* = 0.901), semen volume (*P* = 0.727), and concentration of round cells and neutrophils (*P* = 0.139 and *P* = 0.181). However, the VAS group presented a decrease in sperm concentration (*P* < 0.001), progressive motility (*P* = 0.020) and morphology (*P* < 0.001), as well as decreased total sperm count (*P* < 0.001), motile sperm concentration (*P* < 0.001) and total count of morphologically normal motile sperm (*P* < 0.001) compared to control and VNS (Table 1).

**Table 1:** Comparison of clinical and seminal variables between the control, varicocele with normal semen (VNS) and varicocele with altered semen (VAS) groups.

	**Control** (*n* = 27)		**VNS group** (*n* = 46)		**VAS group** (*n* = 17)		*P*
	Mean ± S.D.	95% CI	Mean ± S.D.	95% CI	Mean ± S.D.	95% CI	
Age	16.2 1.21	15.71–16.66	16.2 ± 1.25	15.78–16.52	16.1 ± 1.27	15.47–16.77	0.984
Abstinence (days)	3.8 ± 1.46	3.18–4.34	3.8 ± 1.23	3.44–4.17	3.9 ± 1.33	3.26–4.63	0.901
Left testicular volume (mL)	18.2 ± 3.82	16.61–19.66	17.4 ± 3.10	16.49–18.35	16.1 ± 4.16	13.92–18.20	0.168
Right testicular volume (mL)	18.9 ± 4.01	17.30–20.48	18.3 ± 3.43	17.28–19.34	17.2 ± 4.05	15.15–19.32	0.363
Semen volume (mL)	2.5 ± 0.87	2.17–2.85	2.4 ± 0.99	2.09–2.67	2.3 ± 1.07	1.73–2.83	0.727
Progressive motility (%)	61.0 ± 8.61^a^	57.61–64.42	60.0 ± 11.76^a^	56.48–63.47	51.7 ± 14.09^b^	44.41–58.89	**0.020**
Non–progressive motility (%)	3.1 ± 1.59^a^	2.44–3.70	3.2 ± 1.92^a^	2.58–3.72	4.8 ± 1.89^b^	3.79–5.74	**0.005**
Immotility (%)	36.1 ± 7.94	32.95–39.23	36.9 ± 10.85	33.65–40.09	43.6 ± 12.99	36.91–50.27	0.050
Sperm concentration (×10^6^/mL)	83.2 ± 50.85^a^	63.06–103.29	81.1 ± 66.25^a^	61.44–100.79	14.4 ± 17.27^b^	5.47–23.23	**<0.001**
Total sperm count (×10^6^)	203.9*	153.88^a^ (112.48–266.35)^†^	139.9*	122.69^a^ (86.03– 208.71)^†^	15.3*	44.80^b^ (5.5–50.30)^†^	**<0.001**
Motile sperm concentration (×10^6^/mL)	50.7 ± 31.25^a^	38.32–63.04	48.1 ± 40.17^a^	36.15–60.01	7.2 ± 8.05^b^	3.08–11.36	**<0.001**
Morphology (%)	9.1 ± 1.92^a^	8.31–9.83	8.6 ± 2.91^a^	7.69–9.42	2.8 ± 1.68^b^	1.90–3.63	**<0.001**
Total count of morphologically normal motile spermatozoa (×10^6^)	10.4*	8.07^a^ (6.51–14.57)^†^	7.7*	10.28^a^ (3.39–13.67)^†^	0.2*	0.37^b^ (0.05–0.42)^†^	**<0.001**
Round cells concentration (×10^6^/mL)	2.0*	1.38 (1.50–2.88	1.5*	2.36 (0.88– 3.24)	0.8*	3.80 (0.45–4.25)	0.139
Neutrophils (×10^6^/mL)	0.0*	0.10 (0.00–0.10)	0.05*	0.24 (0.00 – 0.24)	0.0*	0.15 (0.00 – 0.15)	0.181

Different letters in the same line indicate significant difference (*P*  <0.05) in the *post hoc* Tukey HSD or Tamhane. Statistically significant values are presented in bold.

*Values are presented as median; †Values are presented as interquartile range (Q1–Q3)

### Antioxidants enzyme activity and lipid peroxidation levels

The catalase activity was undetectable in seminal plasma by the employed method. Seminal enzymatic activities of superoxide dismutase (SOD) and glutathione peroxidase (GPx) did not differ between groups (*P* = 0.914 and *P* = 0.497) (Table 2). The VAS group presented an increase of TBARS/10^6^ sperm compared to control and VNS (*P* < 0.001) and TBARS in serum did not differ between groups (*P* = 0.459).

**Table 2:** Seminal enzymatic activities of superoxide dismutase (SOD) and glutathione peroxidase (GPx) Seminal concentrations of thiobarbituric acid reactive substances (TBARS)/10^6^ spermatozoa and serum TBARS levels in control, varicocele with normal semen (VNS) and varicocele with altered semen (VAS) groups. Data are described as mean; standard deviation.

	**Control group** (*n* = 27)		**VNS group** (*n* = 46)		**VAS group** (*n* = 17)		*P***-values**
	Mean ± S.D.	95% CI	Mean±S.D.	95% CI	Mean±S.D.	95% CI	
SOD (IU/mL semen)	75.4 ± 35.58	61.29– 89.44	75.1 ± 29.99	66.20– 84.01	71.6 ± 30.94	55.67–87.48	0.914
GPx (IU/mL semen)	166.3 ± 22.28	157.45–175.08	161.46 ± 23.64	154.44– 168.48	167.0 ± 22.67	156.44–180.31	0.497
TBARS/spermatozoa (ng/10^6^ sperm)	4.35 ± 2.28^a^	3.43–5.27	5.2 ± 3.59^a^	4.17–6.30	69.6 ± 84.44^b^	24.59–114.59	**<0.001**
TBARS serum (ng/mL)	4170.1 ± 528.42	3961.07–4379.14	4337.1 ± 542.63	4176.01–4498.29	4300.1 ± 625.60	3978.46–4621.77	0.459

Significant difference is presented in bold; Different letters in the same line indicate significant difference in the* post hoc* Tukey HSD.

### Correlations

The correlation index between clinical and seminal variables, SOD and GPx activities and lipid peroxidation levels in semen (TBARS/10^6^ sperm) and serum (ng/mL) in the control, VNS and VAS groups are demonstrated in the Table 3. Sperm concentration was negatively correlated to TBARS/10^6^ sptz in all groups: control (r = −0.765, *P* < 0.001), VSN (r = −0.680, *P* < 0.001) and VSA (r = −0.503, *P* = 0.047). Also, Log TMNM was negatively correlated to TBARS/10^6^ sptz in the control group (r = −0.835, *P* < 0.001), in the VNS group (r = −0.791, *P* < 0.001) and in VAS group (r = −0.769, *P* < 0.001). Non-progressive motility (NP) was negatively correlated to GPx activity (r = −0.421, *P* = 0.029) only in the control group. On the other hand, non-progressive motility (NP) was positively correlated to TBARS/10^6^ sptz (r = 0.530, *P* = 0.035) only in the VAS group. TBARS / 10^6^ sptz was negatively correlated to morphology in the control group (r = −0.516, *P* = 0.007) and VNS (r = −0.451, *P* = 0.002). Finally, ejaculate volume was negatively correlated to GPx activity (r = −0.580, *P* = 0.015) only in the VAS group.

**Table 3:** Correlation coefficient between clinical and seminal variables superoxide dismutase (SOD) and glutathione peroxidase (GPx) activity, thiobarbituric acid reactive substances (TBARS)/10^6^ sperm and serum TBARS levels between control, varicocele with normal semen (VNS) and varicocele with altered semen (VAS) groups.

	SOD (UI/mL)			GPx (UI/mL)			TBARS /10^6^ sperm (ng/10^6^ xsptz)			Serum TBARS (ng/mL)		
	Control (*n* = 27)	VSN (*n* = 46)	VSA (*n* = 17)	Control (*n* = 27)	VSN (*n* = 46)	VSA (*n* = 17)	Control (*n* = 27)	VSN (*n* = 46)	VSA (*n* = 17)	Control (*n* = 27)	VSN (*n* = 46)	VSA (*n* = 17)
Left testicular volume (mL)							−0.538	−0.344	–			
Right testicular volume (mL)							−0.56	−0.302	–			
Abstinence (days)										–	−0.35	–
Semen volume (mL)				–	–	−0.58						
Non-progressive motility (%)				−0.421	–	–	–	–	0.53			
Sperm concentration (x 10^6^/mL)							−0.765	−0.68	−0.503			
Morphology (%)							−0.516	−0.451	–			
LOG TMNM (x10^6^)							−0.835	−0.791	−0.769			
SOD (U/mL)										–	−0.411	–

TMNM, total count of morphologically normal motile sperm.

## Discussion

Varicocele, due to several and interrelated mechanisms, may cause testicular dysfunction, compromising the process of spermatogenesis and leading to decreased semen quality and/or sperm function, thus impairing male fertility (Hassanin *et al.* 2018). However, as not every adult with varicocele is infertile, the study of the mechanisms associated with the onset of varicocele-related infertility may increase the understanding of the early mechanisms of testicular injury.

Several mechanisms have been proposed to be involved in the pathophysiology of varicocele, which we can highlight the oxidative stress (Agarwal *et al.* 2009, Robinson *et al.* 2010). Oxidative stress is described as the imbalance between the production of ROS and the antioxidant protection of semen, in favor of the oxidant (Aitken & Krausz 2001). The production of ROS by sperm is a normal physiological process, being important for hyperactivation regulation, acrosome reaction and the fusion between sperm and oocyte. Despite the physiologically normal effect of ROS on the sperm cell, an imbalance between the production and the elimination of these species in the semen causes harmful effects to the sperm. The semen's antioxidant mechanisms, whether enzymatic or non-enzymatic, are responsible for trying to maintain this oxidative balance and avoid such damage (de Lamirande & Gagnon 1993, Aitken *et al.* 1997, de Lamirande *et al.* 1997).

For a better understanding of the role of oxidative stress in the establishment of testicular dysfunction in varicocele, the present study aimed to evaluate if there is an association between semen lipid peroxidation levels and semen quality in adolescents with and without varicocele and the role of seminal enzymatic antioxidants in varicocele-induced oxidative stress. In our study, increased lipid peroxidation levels were observed in the varicocele altered semen (VAS) group compared to the other groups. However, no differences were observed between the groups regarding superoxide dismutase and glutathione peroxidase activities in the seminal plasma and TBARS levels in the serum of adolescents.

It is possible to suggest that the seminal oxidative stress observed in the VAS group can result from the testicular venous stasis caused by varicocele, leading to heat stress and accumulation of toxic metabolites, causing an increase in ROS production (Agarwal *et al.* 2012, Hamada *et al.* 2013, Ritchie & Ko 2021). Moreover, it is important to highlight that the substrate for the seminal lipid peroxidation in this study was exclusively the sperm membrane, given that samples with leukocytospermia were excluded. Therefore, because in the VAS group the sperm concentration is decreased, it is suggested that in this group each sperm is being subjected to lipid peroxidation more intensely than in the other groups.

Although seminal lipid peroxidation levels were increased, no differences were observed between the groups regarding TBARS levels in serum. Furthermore, the enzymatic activities of SOD and GPx in the seminal plasma did not differ between the groups, although the values of TBARS/10^6^ sptz were much greater in the VAS group. Thus, this increased oxidative stress in the VAS group does not seem to be related to a decreased seminal plasma antioxidant capacity, as we had hypothesized, nor to a systemic oxidative stress in adolescents with varicocele. Although some studies have demonstrated a decreased semen total antioxidant capacity (TAC), and SOD, GPx and catalase levels in seminal plasma of men with varicocele (Pasqualotto *et al.* 2008, Mostafa *et al.* 2012), and an increase in the concentration of seminal antioxidants after varicocelectomy (Chen *et al.* 2008), these studies were performed in adult men, not in adolescents. Therefore, we can speculate that the reduction in antioxidant enzyme activity caused by varicocele, can be a process that takes place over time (Andriollo-Sanchez *et al.* 2005, Nguyen-Powanda & Robaire 2020).

In the present study, we observed a negative correlation between TBARS/10^6^ sptz and sperm concentration in all groups; also, a negative correlation between TBARS/10^6^ sptz and morphology was observed in control and VNS groups, but this correlation was lost in the VAS group. This can be explained by a dysregulation in the apoptosis process in the testis of men with varicocele (Fujisawa *et al.* 1999), which may have started in adolescence, as evidenced in a study that showed the presence of proteins with important role in DNA repair and promoting apoptosis (SMG1 and IBP3) exclusively in adolescents with varicocele and altered semen (Zylbersztejn *et al.* 2013).

There was also a positive correlation between non-progressive motility and TBARS/10^6^ sptz only in the VAS group, which again indicates that this group has a greater damage to the sperm membrane when compared to control groups and VNS. Although it is well established that high concentrations of ROS decrease sperm motility (Alahmar 2019), the exact mechanisms through which this occurs are not completely understood. One hypothesis suggests that H_2_O_2_ diffuses through the cell membrane and inhibits the activity of some vital enzymes such as glucose-6-phosphate dehydrogenase (Gomez *et al.* 1996).

The correlation of oxidative stress with sperm concentration, motility, and especially morphology corroborates the finding of a negative correlation between Log TMMN and TBARS/10^6^ sptz in the control group, the VNS group and VAS group. This shows that when these three variables are analyzed together, the correlation with oxidative stress is even stronger.

Shalini and Bansal considered the GPx an oxidative stress marker since its activity seems to be altered in response to a stress (Shalini & Bansal 2005). Accordingly, in the present study, a negative correlation was observed between non-progressive motility (NP) and GPx activity only in the control group, indicating that this GPx response to stress may be limited. The fact that this correlation was lost in the groups with varicocele, and taking into account that the activity of antioxidant enzymes did not differ between the groups, suggests that the presence of the disease can result in a higher testicular stress than the physiological enzymatic responsiveness. This correlation only in the control group could also indicate that the system is in oxidative homeostasis: the GPx would be produced in response to any stress and would be effective in keeping the ideal patterns of motility. Finally, new controlled and carefully designed studies are needed to clarify how oxidative stress and its markers are associated with decreased fertility potential and thereby to identify which adolescents would benefit from early correction of varicocele.

## Conclusion

In conclusion, varicocele leads to seminal oxidative stress that is not caused by decreased activity of antioxidant enzymes. Bearing this in mind, it is important to appropriately address varicocele in order to remove pro-oxidative conditions, rather than to focus on re-establishing antioxidant activity.

## Declaration of interest

The authors declare that there is no conflict of interest that could be perceived as prejudicing the impartiality of the research reported.

## Author contribution statement

V B; D M S Conception, designing, conducting and writing the manuscript. V B; M N Sampling and sperm analyses. M P A; P I Data interpretation and revision of the article. R P B Data interpretation and statistical analysis. All authors read and approved the final manuscript.
